# Supplementary material for: Bundle-specific associations between white matter microstructure and Aβ and tau pathology in preclinical Alzheimer’s disease
Source: eLife. 2021 May 13;10:e62929. doi: 10.7554/eLife.62929 (PMC8169107; doi:10.7554/eLife.62929)
Supplement: Figure 2—source data 1. [file elife-62929-fig2-data1.docx]

**Figure 2- source data 1. Associations between microstructure and Aβ-PET in PREVENT-AD**

| **WHOLE GROUP** |  |  |  |  |  |  |
| --- | --- | --- | --- | --- | --- | --- |
|  | **Anterior cingulum** | | **Posterior cingulum** | | **Uncinate fasciculus** | |
|  | R_partial_ | p-value | R_partial_ | p-value | R_partial_ | p-value |
| Left hemisphere |  |  |  |  |  |  |
| FA_T_ | 0.092 | 0.314 | -0.01 | 0.914 | 0.017 | 0.853 |
| MD_T_ | -0.091 | 0.317 | 0.01 | 0.914 | -0.017 | 0.857 |
| AD_T_ | 0.15 | 0.099 | 0.027 | 0.771 | 0.017 | 0.852 |
| RD_T_ | -0.092 | 0.314 | 0.01 | 0.917 | -0.017 | 0.857 |
| FW | 0.061 | 0.503 | 0.031 | 0.734 | 0.088 | 0.336 |
| Right hemisphere |  |  |  |  |  |  |
| FA_T_ | 0.128 | 0.16 | 0.004 | 0.967 | -0.05 | 0.584 |
| MD_T_ | -0.129 | 0.157 | -0.003 | 0.973 | 0.051 | 0.581 |
| AD_T_ | 0.079 | 0.387 | 0.039 | 0.67 | -0.063 | 0.491 |
| RD_T_ | -0.129 | 0.158 | -0.003 | 0.971 | 0.051 | 0.579 |
| FW | 0.089 | 0.331 | 0.115 | 0.206 | 0.134 | 0.143 |
| **Aβ-POSITIVE** | |  |  |  |  |  |
|  | **Anterior cingulum** | | **Posterior cingulum** | | **Uncinate fasciculus** | |
|  | R_partial_ | p-value | R_partial_ | p-value | R_partial_ | p-value |
| Left hemisphere |  |  |  |  |  |  |
| FA_T_ | -0.13 | 0.563 | **-0.426** | 0.048 | **-0.521** | 0.013 |
| MD_T_ | 0.134 | 0.552 | **0.427** | 0.047 | **0.523** | 0.012 |
| AD_T_ | 0.062 | 0.785 | -0.169 | 0.452 | -0.413 | 0.056 |
| RD_T_ | 0.133 | 0.556 | **0.426** | 0.048 | **0.523** | 0.012 |
| FW | -0.248 | 0.266 | 0.103 | 0.647 | 0.145 | 0.519 |
| Right hemisphere |  |  |  |  |  |  |
| FA_T_ | 0.065 | 0.774 | -0.255 | 0.253 | -0.401 | 0.065 |
| MD_T_ | -0.065 | 0.773 | 0.259 | 0.245 | 0.404 | 0.063 |
| AD_T_ | -0.027 | 0.906 | -0.019 | 0.934 | -0.382 | 0.08 |
| RD_T_ | -0.064 | 0.776 | 0.258 | 0.247 | 0.404 | 0.063 |
| FW | 0.056 | 0.804 | 0.211 | 0.347 | -0.215 | 0.337 |

R_partial_ and p-values from regression models investigating associations between each diffusion measure (average diffusion measure in the bundle; independent variable) and global cortical Aβ pathology across all PREVENT-AD participants (dependent variable) in the top panel and in the Aβ-positive participants only in the bottom panel. Models included age, sex, bundle volume (divided by total intracranial volume) as covariates.

Aβ: beta-amyloid; FA_T_: tissue fractional anisotropy; MD_T_: tissue mean diffusivity; AD_T_: tissue axial diffusivity; RD_T_: tissue radial diffusivity; FW: free-water index
