# Supplementary material for: Bundle-specific associations between white matter microstructure and Aβ and tau pathology in preclinical Alzheimer’s disease
Source: eLife. 2021 May 13;10:e62929. doi: 10.7554/eLife.62929 (PMC8169107; doi:10.7554/eLife.62929)
Supplement: Figure 3—source data 1. [file elife-62929-fig3-data1.docx]

**Figure 3- source data 1. Associations between microstructure and tau-PET in PREVENT-AD**

| **WHOLE GROUP** | |  |  |  |  |  |
| --- | --- | --- | --- | --- | --- | --- |
|  | **Anterior cingulum** | | **Posterior cingulum** | | **Uncinate fasciculus** | |
|  | R_partial_ | p-value | R_partial_ | p-value | R_partial_ | p-value |
| Left hemisphere |  |  |  |  |  |  |
| FA_T_ | -0.009 | 0.921 | -0.034 | 0.713 | 0.088 | 0.336 |
| MD_T_ | 0.009 | 0.92 | 0.033 | 0.718 | -0.088 | 0.336 |
| AD_T_ | 0.032 | 0.727 | -0.057 | 0.529 | 0.085 | 0.352 |
| RD_T_ | 0.009 | 0.923 | 0.033 | 0.716 | -0.088 | 0.335 |
| FW | 0.067 | 0.461 | 0.161 | 0.077 | 0.043 | 0.64 |
| Right hemisphere |  |  |  |  |  |  |
| FA_T_ | -0.056 | 0.542 | -0.028 | 0.758 | -0.079 | 0.384 |
| MD_T_ | 0.056 | 0.54 | 0.029 | 0.755 | 0.08 | 0.38 |
| AD_T_ | -0.043 | 0.641 | -0.034 | 0.712 | -0.057 | 0.532 |
| RD_T_ | 0.056 | 0.541 | 0.029 | 0.754 | 0.08 | 0.381 |
| FW | 0.065 | 0.474 | 0.117 | 0.2 | 0.147 | 0.107 |
| **TAU-POSITIVE** | |  |  |  |  |  |
|  | **Anterior cingulum** | | **Posterior cingulum** | | **Uncinate fasciculus** | |
|  | R_partial_ | p-value | R_partial_ | p-value | R_partial_ | p-value |
| Left hemisphere |  |  |  |  |  |  |
| FA_T_ | -0.336 | 0.147 | **-0.541** | 0.014 | 0.109 | 0.647 |
| MD_T_ | 0.34 | 0.142 | **0.539** | 0.014 | -0.11 | 0.645 |
| AD_T_ | -0.183 | 0.439 | **-0.484** | 0.03 | 0.023 | 0.924 |
| RD_T_ | 0.339 | 0.143 | 0.54 | 0.014 | -0.109 | 0.647 |
| FW | 0.184 | 0.437 | 0.324 | 0.164 | -0.059 | 0.806 |
| Right hemisphere |  |  |  |  |  |  |
| FA_T_ | -0.323 | 0.165 | -0.424 | 0.062 | -0.204 | 0.388 |
| MD_T_ | 0.33 | 0.156 | 0.428 | 0.059 | 0.209 | 0.376 |
| AD_T_ | -0.119 | 0.617 | -0.241 | 0.307 | -0.215 | 0.362 |
| RD_T_ | 0.328 | 0.158 | 0.428 | 0.06 | 0.21 | 0.375 |
| FW | 0.24 | 0.308 | 0.28 | 0.232 | 0.137 | 0.565 |

R_partial_ and p-values from regression models investigating associations between each diffusion measure (average diffusion measure in the bundle; independent variable) and entorhinal tau across all PREVENT-AD participants (dependent variable) in the top panel and in the tau-positive participants only in the bottom panel. Models included age, sex, bundle volume (divided by total intracranial volume) as covariates.

FA_T_: tissue fractional anisotropy; MD_T_: tissue mean diffusivity; AD_T_: tissue axial diffusivity; RD_T_: tissue radial diffusivity; FW: free-water index
