# Supplementary material for: Bundle-specific associations between white matter microstructure and Aβ and tau pathology in preclinical Alzheimer’s disease
Source: eLife. 2021 May 13;10:e62929. doi: 10.7554/eLife.62929 (PMC8169107; doi:10.7554/eLife.62929)
Supplement: Figure 4—source data 1. [file elife-62929-fig4-data1.docx]

**Figure 4- source data 1. Associations between microstructure and Aβ-PET in DIAN**

| **WHOLE GROUP** | |  |  |  |  |  |
| --- | --- | --- | --- | --- | --- | --- |
|  | **Anterior cingulum** | | **Posterior cingulum** | | **Uncinate fasciculus** | |
|  | R_partial_ | p-value | R_partial_ | p-value | R_partial_ | p-value |
| Left hemisphere |  |  |  |  |  |  |
| FA_T_ | **-0.272** | 0.016 | -0.097 | 0.397 | 0.027 | 0.812 |
| MD_T_ | **0.276** | 0.015 | 0.099 | 0.39 | -0.023 | 0.842 |
| AD_T_ | 0.013 | 0.913 | -0.024 | 0.837 | 0.153 | 0.181 |
| RD_T_ | **0.275** | 0.015 | 0.099 | 0.391 | -0.025 | 0.825 |
| FW | -0.091 | 0.43 | -0.235 | 0.039 | 0.088 | 0.442 |
| Right hemisphere |  |  |  |  |  |  |
| FA_T_ | -0.194 | 0.089 | -0.183 | 0.108 | -0.05 | 0.664 |
| MD_T_ | 0.2 | 0.079 | 0.186 | 0.103 | 0.05 | 0.667 |
| AD_T_ | 0.078 | 0.495 | -0.092 | 0.426 | -0.025 | 0.826 |
| RD_T_ | 0.198 | 0.082 | 0.186 | 0.103 | 0.05 | 0.665 |
| FW | -0.189 | 0.097 | -0.037 | 0.75 | -0.133 | 0.246 |
| **Aβ-POSITIVE** |  |  |  |  |  |  |
|  | **Anterior cingulum** | | **Posterior cingulum** | | **Uncinate fasciculus** | |
|  | R_partial_ | p-value | R_partial_ | p-value | R_partial_ | p-value |
| Left hemisphere |  |  |  |  |  |  |
| FA_T_ | **-0.405** | 0.021 | -0.208 | 0.253 | -0.084 | 0.649 |
| MD_T_ | **0.405** | 0.021 | 0.209 | 0.252 | 0.113 | 0.538 |
| AD_T_ | -0.307 | 0.088 | -0.175 | 0.338 | 0.325 | 0.069 |
| RD_T_ | **0.406** | 0.021 | 0.209 | 0.251 | 0.103 | 0.575 |
| FW | -0.135 | 0.46 | -0.13 | 0.479 | 0.043 | 0.816 |
| Right hemisphere |  |  |  |  |  |  |
| FA_T_ | **-0.461** | 0.008 | **-0.344** | 0.054 | -0.193 | 0.289 |
| MD_T_ | **0.459** | 0.008 | **0.344** | 0.054 | 0.214 | 0.24 |
| AD_T_ | -0.221 | 0.224 | -0.339 | 0.057 | 0.158 | 0.389 |
| RD_T_ | **0.460** | 0.008 | **0.345** | 0.053 | 0.208 | 0.254 |
| FW | -0.253 | 0.162 | -0.091 | 0.622 | 0.072 | 0.696 |

R_partial_ and p-values from regression models investigating associations between each diffusion measure (average diffusion measure in the bundle; independent variable) and global cortical Aβ pathology across all DIAN participants (dependent variable) in the top panel and in the Aβ-positive participants only in the bottom panel. Models included age, sex, bundle volume (divided by total intracranial volume) as covariates.

Aβ: beta-amyloid; FA_T_: tissue fractional anisotropy; MD_T_: tissue mean diffusivity; AD_T_: tissue axial diffusivity; RD_T_: tissue radial diffusivity; FW: free-water index
